# Benign and Suspicious Ovarian Masses—MR Imaging Criteria for Characterization: Pictorial Review

**DOI:** 10.1155/2012/481806

**Published:** 2012-03-22

**Authors:** A. L. Valentini, B. Gui, M. Miccò, M. C. Mingote, A. M. De Gaetano, V. Ninivaggi, L. Bonomo

**Affiliations:** Department of Bioimaging and Radiological Sciences, Catholic University of Rome, Largo A Gemelli, 8, 00168 Rome, Italy

## Abstract

Ovarian masses present a special diagnostic challenge when imaging findings cannot be categorized into benign or malignant pathology. Ultrasonography (US), Computed Tomography (CT), and Magnetic Resonance Imaging (MRI) are currently used to evaluate ovarian tumors. US is the first-line imaging investigation for suspected adnexal masses. Color Doppler US helps the diagnosis identifying vascularized components within the mass. CT is commonly performed in preoperative evaluation of a suspected ovarian malignancy, but it exposes patients to radiation. When US findings are nondiagnostic or equivocal, MRI can be a valuable problem solving tool, useful to give also surgical planning information. MRI is well known to provide accurate information about hemorrhage, fat, and collagen. It is able to identify different types of tissue contained in pelvic masses, distinguishing benign from malignant ovarian tumors. The knowledge of clinical syndromes and MRI features of these conditions is crucial in establishing an accurate diagnosis and determining appropriate treatment. The purpose of this paper is to illustrate MRI findings in neoplastic and non-neoplastic ovarian masses, which were assessed into three groups: cystic, solid, and solid/cystic lesions. MRI criteria for the correct diagnosis and characteristics for differentiating benign from malignant conditions are shown in this paper.

## 1. Introduction

Ovarian masses present a special diagnostic challenge when imaging findings cannot be categorized into benign or malignant pathology. Ultrasonography (US), Computed Tomography (CT), and Magnetic Resonance Imaging (MRI) are currently used to evaluate ovarian tumors. US is the first-line imaging investigation for suspected adnexal masses helping in detection and characterization of ovarian tumors [1]. US morphological analysis of adnexal masses is usually accurate for identifying low risk or high risk lesions [2]. The most important morphological features for high risk ovarian masses include (a) solid/cystic or solid lesions with a maximum diameter greater than 4 cm; (b) the presence of irregular, nonfatty, solid vascularized areas greater than 28 mm in diameter [3]; (c) the presence of papillary projection (vegetation) and thick wall and septa greater than 3 mm in a cystic lesion [4]. Color Doppler US helps the diagnosis identifying vascularized components within the mass [5]. Though US can be inconclusive. An adnexal mass is defined as indeterminate on US when it cannot be confidently placed into either the benign or malignant category, even after thorough interrogation including Doppler assessment, or for which the site of origin, from the ovary, uterus or another pelvic structure, remains to be established [6]. CT is commonly performed in preoperative evaluation of a suspected ovarian malignancy, but it exposes patients to radiation. When US findings are nondiagnostic or equivocal, MRI can be a valuable problem solving tool, an adjunctive modality for evaluating adnexal lesions, useful to give also surgical planning information without radiation exposure [7]. MRI is well known to provide accurate information about hemorrhage, fat, and collagen [6]. It is able to identify different types of tissue contained in pelvic masses, distinguishing benign from malignant ovarian tumors, with an overall accuracy of 88% to 93% [8]. Benign ovarian diseases can simulate malignancies. The knowledge of clinical syndromes and MRI features of these conditions is crucial in establishing an accurate diagnosis and determining appropriate treatment.

The purpose of this paper is to illustrate MRI findings in neoplastic and nonneoplastic ovarian masses, separated into three groups: cystic, solid, and solid/cystic lesions. MRI criteria for the correct diagnosis and characteristics for differentiating benign from suspicious conditions are shown in this paper.

## 2. MRI Technical Suggestions

Patients undergoing MRI examination for evaluating an ovarian mass must fast for 3-4 hours and receive an antispasmodic drug, intramuscularly, 10 minutes before MRI, in order to reduce bowel peristalsis, also to improve visualization of the adnexa and peritoneal surfaces. For an adequate pelvic MRI evaluation, images must be obtained minimum in two planes. T1-weighted spin-echo images in the axial plane and T2-weighted fast spin-echo images obtained in the axial, sagittal, and coronal planes are usually employed to evaluate uterus, adjacent organs, pelvic floor, and broad ligaments, providing panoramic “morphological” view of the pelvis. Acquisition of T1- and T2-weighted images is fundamental for pelvic anatomy and tissue characterization [9–11].

T1-weighted images with a selective chemical fat-suppression technique in the axial plane are useful to identify blood and fat tissue [11–14]. T2-weighted images with a selective chemical fat-suppression technique can be usefully employed to better identify inflammation changes or edema. 3D fat-saturated T1-weighted Spoiled Gradient Echo (SPGR) images after intravenous contrast material help detection of solid components into the mass, also improving detection of peritoneal and omental implants [15, 16]. The use of small fields of view (20 cm), high-resolution matrixes (256 × 256), and thin sections (4 mm) improves the delineation of papillary projections into cystic lesions. A body axial T2-weighted sequence for evaluating retroperitoneal space can also be used to complete the pelvis evaluation [8].

Several types of tissue and fluid characterizing an ovarian mass can be distinguished at MRI on the basis of their signal intensity [17]. Cystic lesions demonstrate low signal intensity on T1-weighted images and very high signal intensity on T2-weighted images. Solid portions are characterized by relative high hyperintensity on T2-weighted images. Fat, hemorrhage, and some high-viscosity, mucin-containing lesions have high signal intensity on T1-weighted MRI. Fibrosis or smooth muscle has low or intermediate signal intensity on T1-weighted MRI and low signal intensity on T2-weighted images [6].

## 3. Nonneoplastic Ovarian Conditions

### 3.1. Cystic Lesions

#### 3.1.1. Polycystic Ovary Syndrome

Polycystic ovary syndrome (PCOS) affects approximately 20% of premenopausal women and is one of the most common endocrinopathies of women [18]. The follicles in PCOS are typically smaller in size than those in normal ovaries. US imaging features include 10 or more follicles with a diameter of 2 to 8 mm, arranged around an echo dense central stroma, with a threshold stroma/area (S/A) ratio of 0.34 (calculated as ovarian stromal area to the total area of the ovary). This S/A ratio has been described as having sensitivity and a specificity of 100% [19].

Typical MRI features include enlarged ovaries, with a central stroma and multiple small follicles located peripherally [20, 21]. Enlargement of the central ovarian stroma usually shows low intensity on T2-weighted images. The role of MRI in this condition is excluding other hormone-stimulating ovarian disorders (like Granulosa cell tumor, Sclerosing stromal tumor, and Sertoli-Leydig cell tumor, Steroid cell tumor) by carefully evaluating the presence of solid lesions within the mass [7]. These conditions generally appear as a well-defined, heterogeneous enhancing solid mass with internal cyst or areas of intracellular lipids; they can be associated with endometrial abnormalities [4].

#### 3.1.2. Endometrioma

**Figure 1:**
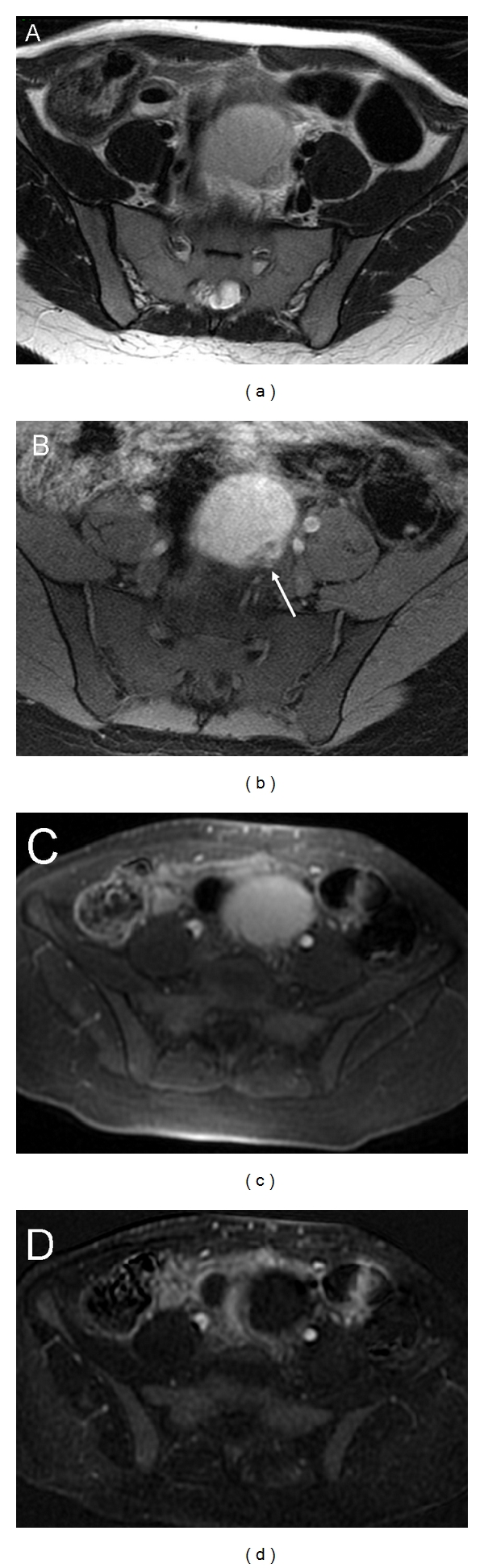
Endometrioma. Axial T2-weighted image (a) shows an ovarian cystic mass with intermediate signal intensity. Axial T1 fat suppression weighted image (b) confirms the haemorrhagic nature of the cyst (endometrioma). Note a mural nodule located posterior on the left side of the wall (b, arrow). 3D fat-saturated T1-weighted Spoiled Gradient Echo images before (c) and after intravenous contrast injection with subtraction of precontrast and postcontrast sequences (d) demonstrate the lack of enhancement of the mural nodule due to blood clot.

Endometrioma occur as a result of extrauterine implantation of endometrial epithelium. The typical US appearance is a complex cystic ovarian mass with homogenous low-level internal echoes and thick wall which is usually hypovascular at color Doppler evaluation. US sensitivity (90.4%) in diagnosing endometrioma is as high as MRI sensitivity (90%); however MRI is usually performed after the US because other foci of endometriosis located in the pelvis and subpelvic spaces can be associated and for excluding malignant degeneration [22, 23]. MRI is able to differentiate endometrioma from endometrioid adenocarcinoma and clear-cell adenocarcinoma that can develop within endometrial cysts, with an incidence of 0.6–1.0% [24]. In these cases, MRI demonstrates solid components in the cystic wall clearly enhancing in postcontrast images. Subtraction images of precontrast and postcontrast sequences are useful when the enhancement evaluation is difficult due to the hyperintensity of endometrial cysts on T1-weighted images (Figure 1). Endometrioma appears on MRI images as high intensity on T1-weighted images and relatively low intensity lesions on T2-weighted images (“shading” effect) manifesting as gradual loss of signal of dependent layering contents [13, 25]. MRI has been reported to have a sensitivity of 68–90% and specificity of 83–98% for the diagnosis of endometrial cysts [13, 26]. The presence of multiple, bilateral lesions add specificity to the diagnosis of endometriosis [19]. Fat saturation technique is highly used to differentiate endometrioma from fatty lesions, also showing high intensity on T1-weighted images, but with loss of signal on saturated T1w images.

#### 3.1.3. Peritoneal Inclusion Cyst

Peritoneal inclusion cyst is defined as fluid accumulation due to adhesions between ovaries and peritoneal after a pelvic surgery or an inflammatory disease. On MRI, this lesion is typically a unilocular or multilocular cystic mass ranging in size from few millimeters to 20 cm or more. Characteristic MRI findings are cystic wall outlined by the pelvic wall, pelvic organs, and bowel loops [7]. Presence of normal ovary entrapped centrally within the lesion creating a “spider in a web” appearance is a suggestive finding. No mural nodules or enhancing solid components are seen [19].

### 3.2. Solid and Solid/Cystic Lesions

#### 3.2.1. Adnexal Torsion

**Figure 2:**
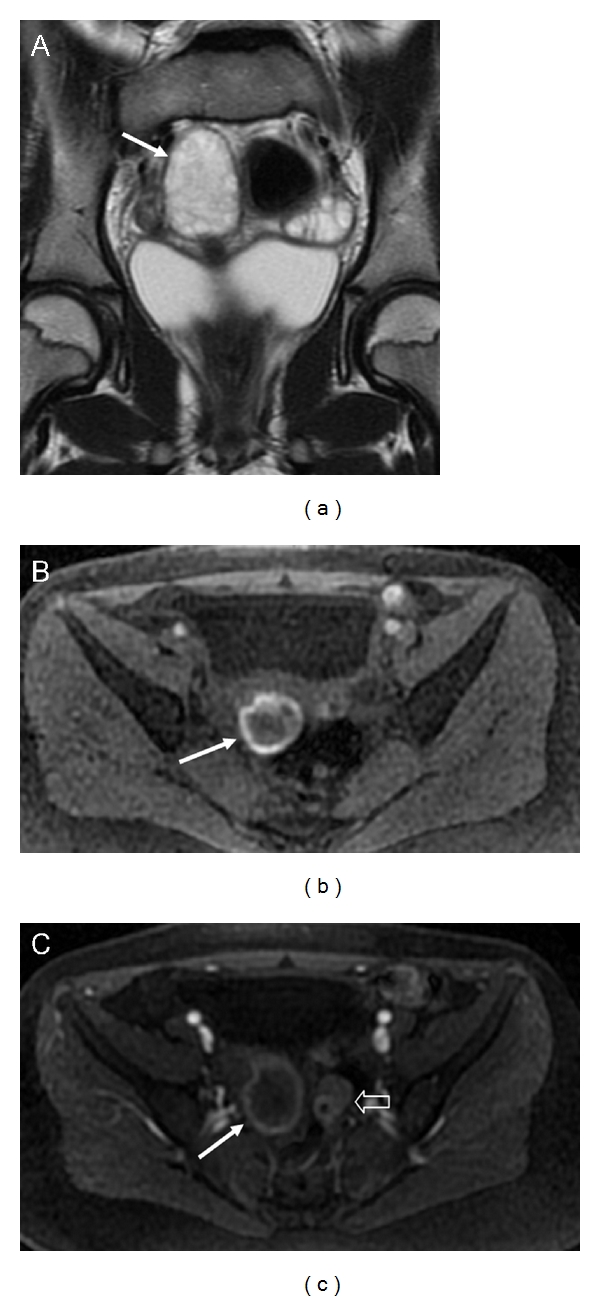
Torsion of right ovary. Coronal T2-weighted image (a) shows an enlarged right ovary with numerous small peripheral cysts representing displaced follicles (arrow). The right ovary displays a peripheral annular hyperintensity on fat suppressed T1-weighted image which is indicative of hemorrhagic infarction (b, arrow). 3D fat-saturated T1-weighted Spoiled Gradient Echo image demonstrate the lack of enhancement of ovarian parenchyma (c, white arrow). Note: normal left ovary with small normal follicle (c, void arrow).

Ovarian torsion most commonly occurs in the first 3 decades of life and frequently involves the ovary and corresponding fallopian tube. It is more often associated with an ovarian cyst or mass (approximately in 50% of cases) [27]. US usually shows an enlarged ovary with follicles distributed peripherally [28]. Doppler US improves diagnostic possibilities of US showing the lack of flow in the twisted ovary. However, torsion may occur with incomplete vascular obstruction. MRI can achieve a more adequate diagnosis showing typical findings as the ovary drawn to the uterus, deviation of the uterus to the side of torsion, straight vessels draping around the lesion, absence of enhancement or massive ovarian edema in the case of intermittent torsion, which is shown as diffuse high signal intensity on T2-weighted images. When hemorrhagic necrosis occurs, the lesion may exhibit high intensity on T1-weighted images [29, 30] (Figure 2).

#### 3.2.2. Pelvic Inflammatory Disease (PID)

Pelvic Inflammatory Disease (PID) is a common condition among women in reproductive age and sexual activity. PID is usually clinically and laboratory diagnosed (pain, fever, and leukocytosis), but imaging studies are performed in patients with uncertain diagnosis, or whose not responding to therapy.

**Figure 3:**
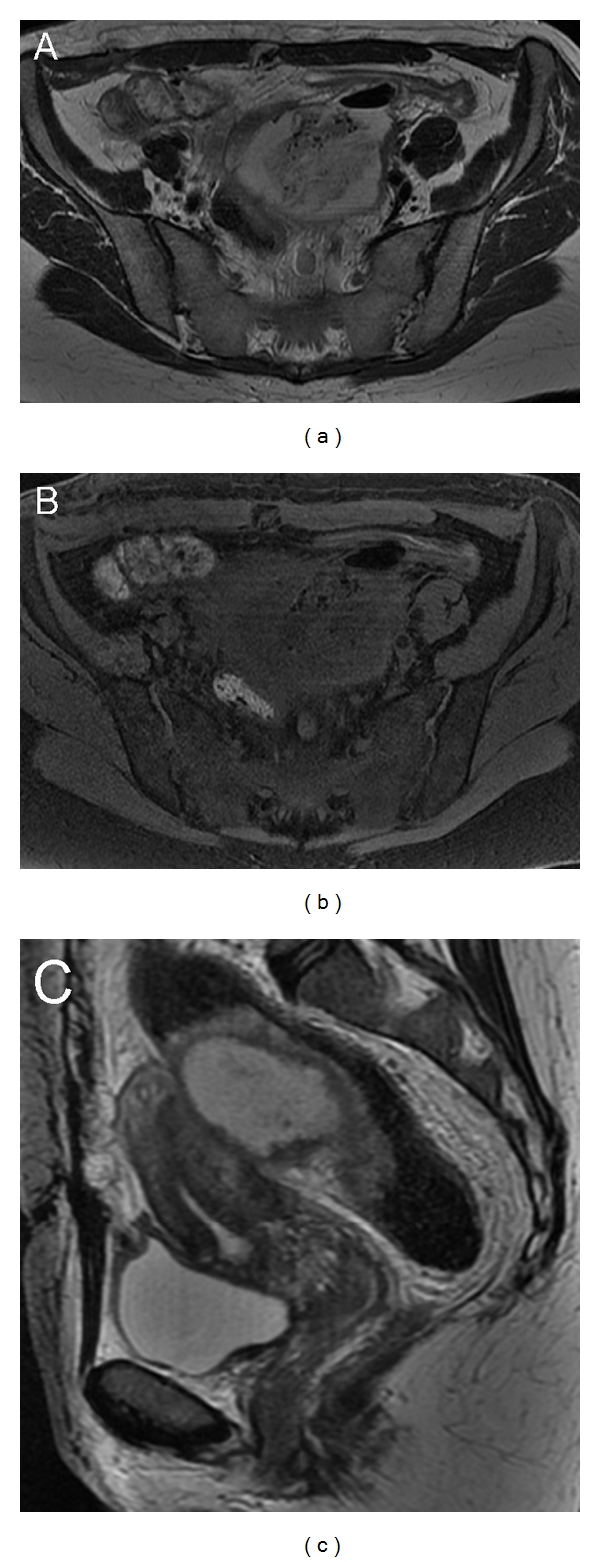
Pelvic inflammatory disease. Left recurrent tuboovarian abscess. Axial T2-weighted image (a) shows a thick wall, complex, heterogeneously hyperintense, fluid-containing adnexal mass, with internal debris and gas bubbles, that represents the most specific sign of an abscess. On axial T1-weighted with fat suppression image (b) there is no signs of hemorrhagic contents within the mass. The sagittal T2-weighted image (c) demonstrates the involvement of bowel loop characterized by the thickening of the anterior rectal wall.

Hydrosalpinx is most often seen on US images as a hypoechoic adnexal mass, with a thick vascularized wall ring, associated with free fluid in the “cul-de-sac”. In emergency, CT is usually requested when US findings are indeterminate [31, 32]. However MRI can be used in mediate urgency showing 95% sensitivity and 89% specificity in the tuboovarian abscess diagnosis [33]. On MRI, a tubo-ovarian abscess is characterized by mild signal intensity on T1-weighted and high signal intensity on T2-weighted images. An irregular thick wall, markedly enhancing after contrast medium administration, and stranding in the surrounding fat planes are characteristic easy to be recognized. In patients with long-term use of intrauterine devices, actinomycetes are a frequent cause of chronic inflammation. On MRI, an ill-defined lesion border resulting from trans fascial inflammatory spread may simulate a malignancy involving the ovary. Relatively low intensity on T2-weighted images representative of fibrosis may be an important tool in the accurate diagnosis of this condition [33, 34] (Figure 3).

## 4. Neoplastic Benign Conditions

### 4.1. Cystic Tumors

#### 4.1.1. Mature Cystic Teratoma (Dermoid Cyst)

Mature cystic teratoma is the most common ovarian tumor (26–44% of all ovarian masses). Incidence picks at 30 years old, generally is found in asymptomatic patients, 90% unilaterally. This benign tumor consists of ectodermal tissue predominantly and is lined with keratinized squamous epithelium and skin appendages. MRI affords a specific diagnosis by demonstrating fat tissue signal which is usually demonstrated as high intensity, the same as subcutaneous fat, on T1-weighted and T2-weighted images. A selective T1-weighted fat suppression technique enables differentiation of cystic teratoma from hemorrhagic adnexal processes such as endometrial cyst. In cases of atypical dermoid cysts with scarce lipid components, chemical shift sequence has been reported to be useful to detect intracellular or microscopic lipid. Other MRI findings for cystic teratomas includes layering or floating debris, soft-tissue protuberances (Rokitansky nodules or dermoid plugs), and low-signal-intensity teeth. MRI is also collaborative in identifying complications associated with dermoid cysts, such as torsion, rupture, and malignant transformation [35].

**Figure 4:**
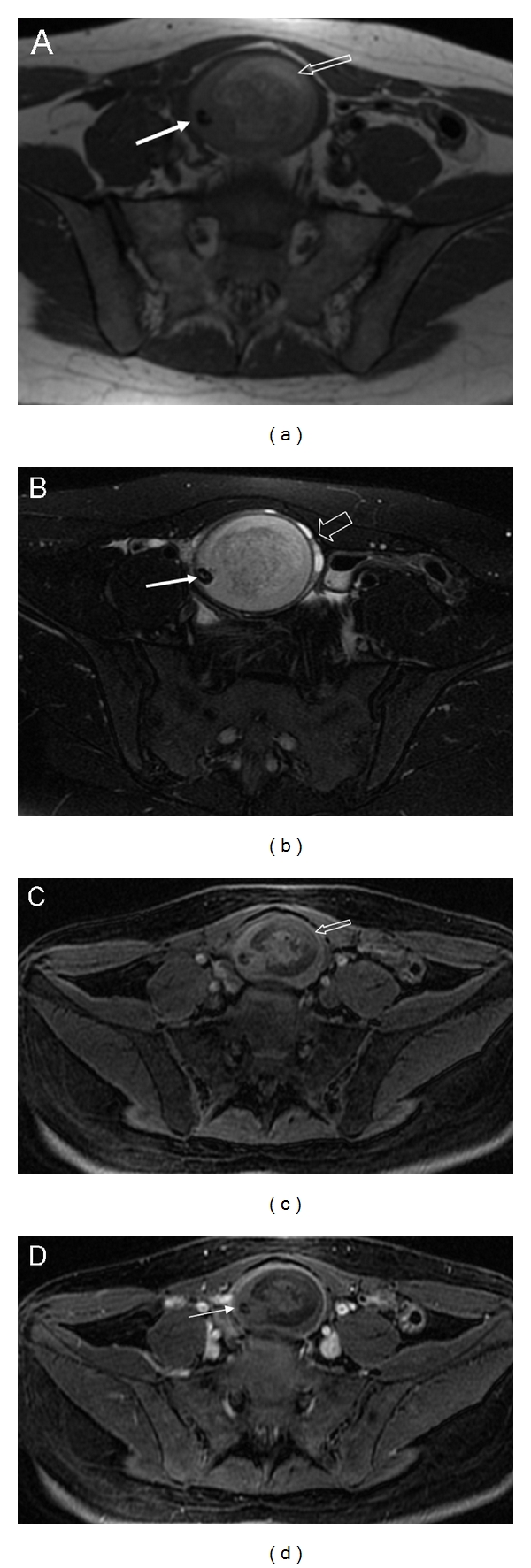
Dermoid cyst with small amount of fat. Axial T1- (a) and T2-weighted images with fat suppression (b) show a complex cystic round lesion with a hypointense nodule (a, b, white arrow) on the right side arising from the right ovary. Observe ovarian parenchyma with small follicles (b, void arrows). A linear T1 hyperintense portion is located on the upper part of the cyst (a, void arrow). The corresponding axial fat suppressed T1 weighted image shows the vanishing of the hyperintense part (c, arrow) confirming the presence of small amount of fat. 3D fat saturated T1-weighted Spoiled Gradient Echo image (d) demonstrates the poor enhancement of mural protrusion corresponding to Rokitansky nodule (arrow).

 Malignant transformation is rare and occurs in approximately 2% of mature cystic teratoma. Invasive squamous carcinomas are the most common malignancy arising in cystic teratoma. The age of patients has a wide range; however, postmenopausal women are most predominantly affected. The tumor tends to spread by direct invasion. The use of intravenous contrast material may be helpful in assessing the malignant solid components (Figure 4).

#### 4.1.2. Cystadenomas

**Figure 5:**
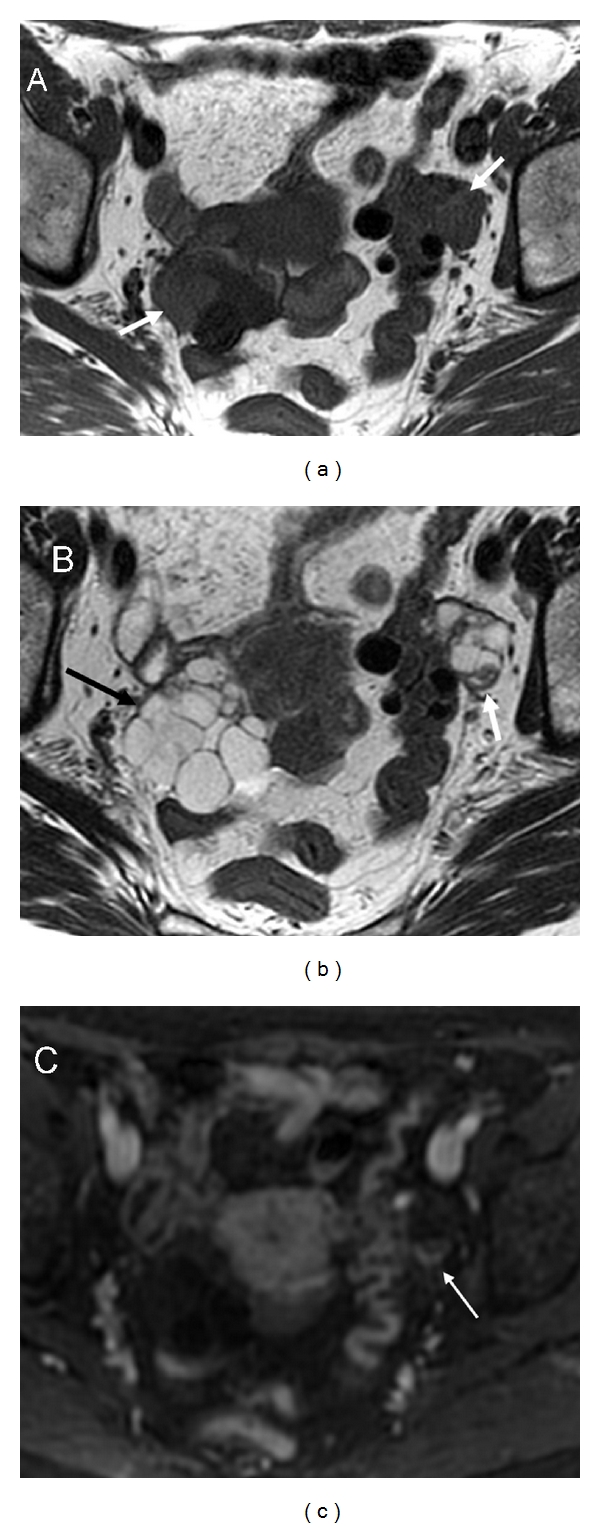
Bilateral serous cystoadenoma. Axial T1- (a, white arrows) and T2-weighted images (b, arrows) demonstrate bilateral multicystic ovarian masses. The cystic loculi are small, multiple, separated by thin septa and characterized by homogeneous low signal intensity on T1 and high intensity on T2-weighted images. Within the left mass there is a posterior small papillary mural projection (b, white arrow). After contrast media administration, 3D fat-saturated T1-weighted Spoiled Gradient Echo image (c, arrow) demonstrate enhancement of the mural projection. This is a highly suspicious sign of malignancy. Histology reports left borderline ovarian serous cystoadenoma and right ovarian serous cystoadenoma.

**Figure 6:**
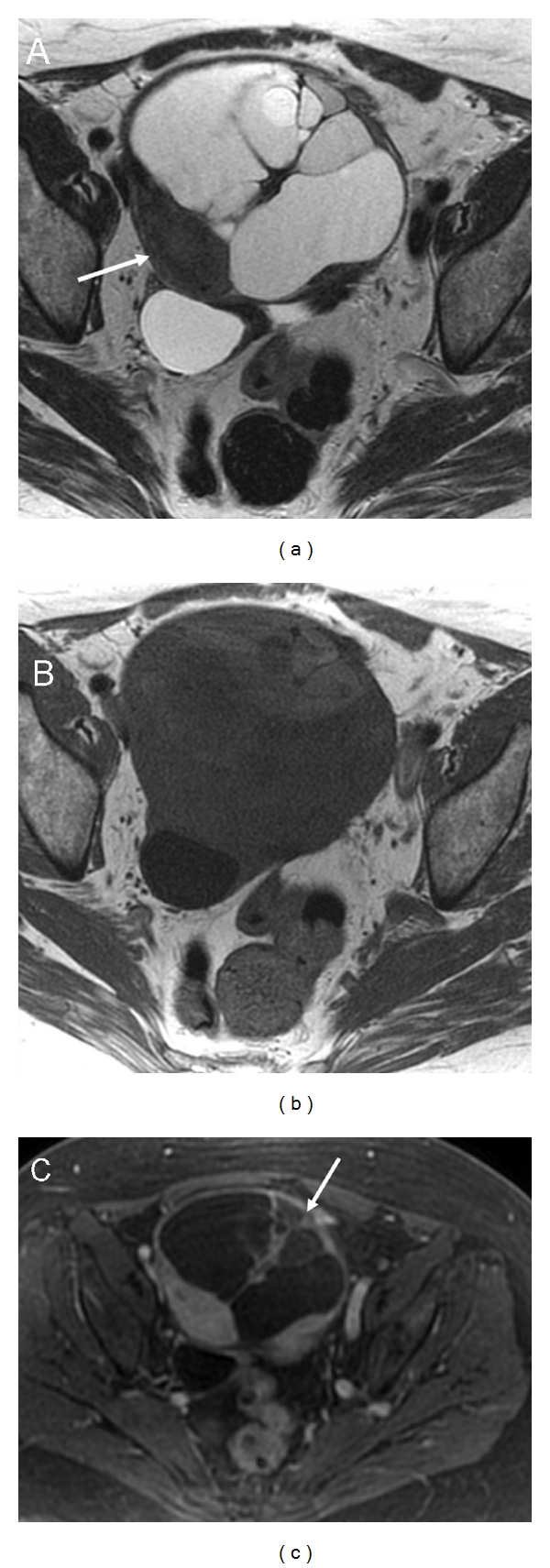
Brenner tumour with mucinous portion. Collision tumour. Axial T2- (a) and T1-weighted (b) images demonstrate an adnexal multilocular cystic mass with thin septations and a solid posterior hypointense portion (a, arrow). The cystic portion presents multiple loculi with different contents characterized by different signal intensity. The septations and the solid portion present an homogeneous and delayed enhancement on 3D fat-saturated T1-weighted Spoiled Gradient Echo images (c, arrow). The interface between the solid and the cystic mass is regular.

Cystadenomas are true cystic epithelial ovarian neoplasm. Serous cystadenomas are common and account for approximately 25% of benign ovarian neoplasm. Incident picks between 20 and 50 years old, bilaterally in 12–23% of cases. The lining of the cyst is flat or may have small papillary projections. The typical MRI appearance of serous cystadenoma is a unilocular cyst with a thin wall (Figure 5). The presence of blood can vary to heterogeneous signal intensity. Mucinous cystadenomas are common and account for approximately 41% of benign ovarian neoplasms. In contrast to serous tumors, only 2%–5% of cases are bilateral [35]. Mucinous cystadenomas are multilocular cysts, larger than serous, containing gelatinous material or fluid of various viscosities. Therefore, the loculi of the tumors often show various signal intensities on both T1- and T2-weighted images (“stained-glass appearance”) (Figure 6). They rarely appear as unilocular cysts. Presence of thick wall or septa may suggest borderline lesions while the presence of solid components suggests carcinoma (Figure 5(c)).

### 4.2. Solid and Solid/Cystic Tumors

#### 4.2.1. Fibroma, Thecoma, and Fibrothecoma

They are the most common solid benign tumors of the ovary. The term fibrothecoma is sometimes applied to these tumors because of their histological overlap. Fibrothecomas are categorized as sex-cord-stromal tumors and are rarely malignant. They are composed of oval or round cells forming variable amounts of collagen. Fibrothecomas can show mixed low to high signal intensity on T2-weighted images. They are more frequently observed during menopause age and sometimes estrogenic stimuli may be associated with endometrial pathology [35]. Because of its histological components, fibromas show predominantly low signal intensity on T2-weighted images and intermediate signal intensity on T1-weighted MRI. On the other hand, edema and cystic formations are relatively common pathologic findings in the tumor because of necrosis. Ovarian fibromas can be infrequently associated with ascites and pleural effusion (Meigs Syndrome). Ovarian fibromatosis, which is a rare benign condition, may cause menstrual abnormalities in young women. This condition can manifest as bilateral ovarian masses with predominant fibrous components, which show low intensity on T2-weighted images. The presence of entrapped follicle between the fibrous tissue may be an important differentiating feature of this condition from ovarian fibromas [7]. Differential diagnosis includes non-degenerated subserosal uterine leiomyoma. Splaying of the uterine myometrium to the mass and vascular signal voids between the uterus and the mass (flow void sign) indicate uterine leiomyoma [35].

#### 4.2.2. Sclerosing Stromal Tumors

Sclerosing stromal tumors are rare, benign, sex-cord stromal tumors that occur predominantly in young women in their second and third decade of life. On MRI, these tumors consist of hyperintense cystic components and heterogeneous solid components with intermediate to high signal intensity on T2-weighted images. Dilated vessels may be observed around the tumor, suggesting tumor hypervascularity. On dynamic contrast-enhanced MRI, the solid component shows early enhancement, which can be a diagnostic clue of the tumor [7].

#### 4.2.3. Brenner Tumor

Brenner tumor is an uncommon surface epithelial tumor that accounts for about 2% of ovarian neoplasms [19]. This tumor can be associated with other cystic ovarian neoplasms, such as cystadenomas, in 30% of cases (Figure 6). On MRI Brenner tumor typically exhibits distinct low intensity on T2-weighted images due to solid components. It is possible to find a characteristic amorphous calcification within the solid components. If present, it is characteristic. However MRI may be limited for detection of calcifications. In these cases, the combination of calcifications demonstrated by ultrasonography or computed tomography and low intensity on T2-weighted MRI strongly suggests the diagnosis of Brenner tumor [35].

#### 4.2.4. Cystadenofibroma

Cystadenofibroma is an uncommon epithelial tumor, which is pathologically characterized by dense fibrous tissue in association with one or many locules. On MRI, these tumors typically manifest as a complex cystic mass associated with solid components of distinct low intensity on T2-weighted images [5, 7, 19].

#### 4.2.5. Struma Ovarii

If the dermoid cyst is composed only of mature thyroid tissue received the name of “struma ovarii.” Typically presented as a multilocular cyst with heterogeneous signal intensity in the locules also known as “stained-glass appearance” on MRI. Thyroid colloid in the cystic space exhibits low intensity on both T1- and T2-weighted images. Most of the cases present fat tissue component being helpful in the final diagnosis [7].

## 5. Discussion and Conclusion

When an adnexal mass is observed on US examination, MRI can be usefully employed to confirm or refute the benign nature of the lesion because the property of tissue characterization. MRI is an effective second confirmatory test also helpful for problem solving since MRI criteria for ovarian malignancy are clearly established. Features suggestive of malignancy (Table 1) include demonstration of solid, solid/cystic enhancing masses (greater than 4 cm in maximum diameter) with papillary projections and irregular thick wall and septa (greater 3 mm) into a cystic lesion (the number of septa and the number and dimension of the vegetations can be suggestive of malignancy). Secondary feature includes the presence of necrosis in a solid mass and intratumoral hemorrhage. Heterogeneous and early enhancement pattern can be suggestive of malignancy. Finally, the ancillary criteria of involvement of pelvic organs or the sidewall, ascites, and lymphadenopathy should be carefully evaluated to distinguish benign from malignant disease [5].

US is an operator-dependent technique, less panoramic than MRI which better identify fat tissue and blood products, that are the main components of most frequent ovarian masses. In addition, MRI dynamic imaging with gadolinium chelates appears promising for problem solving [36]. Combination of US and MRI minimizes the risk of misdiagnosing a malignant mass as benign. Especially in younger women, in whom exposure to ionizing radiation should be as low as possible to preserve fertility. US remains the primary imaging modality in ovarian masses but MRI enables a specific diagnosis to be made for certain pathologic types and has greater specificity in the diagnosis of malignancy with 91–95% accuracy from differentiating benign from malignant adnexal tumors [2, 35]. MRI should be used for characterization of ovarian masses when US results are indeterminate or equivocal, especially when tumor markers are normal or in young patients when conservative surgery is suggestive.

## Figures and Tables

**Table 1:** MRI Criteria for benign and suspicious adnexal lesions.

	Benign	Malignant
Principal criteria		
Size	<4 cm	>4 cm
Solid components	No	Solid part of the mass with heterogeneous enhancement pattern
Cystic mass	Simple	With vegetation and internal structures
Thickness of wall or septa	<3 mm	>3 mm
Lobulated mass	No	Yes
Calcifications	Wall of cyst, dense	Tiny amorphic
Necrosis	No	Yes
Papillary projections	No	Yes, with heterogeneous enhancement pattern
Tumor vessels	No	Yes, with heterogeneous enhancement pattern

Additional criteria		
Lymph nodes	Normal (short axis <1 cm)	Enlarged (short axis >1 cm)
Peritoneal implants	No	Yes

## References

[1] Pierce N, Narayanan P, Sahdev A, Reznek R, Rockall A (2008). Ovarian lesions pose diagnostic dilemmas. *Diagnostic Imaging Europe*.

[2] Spencer JA, Ghattamaneni S (2010). MR imaging of the sonographically indeterminate adnexal mass. *Radiology*.

[3] Bouic-Pagès E, Perrochia H, Mérigeaud S, Giacalone PY, Taourel P (2009). Corrélations anatomopathologiques: IRM des tumeurs ovariennes primitives. *Journal de Radiologie*.

[4] Sohaib SAA, Reznek RH (2007). MR imaging in ovarian cancer. *Cancer Imaging*.

[5] Jeong YY, Outwater EK, Kang HK (2000). From the RSNA refresher courses: imaging evaluation of ovarian masses. *Radiographics*.

[6] Spencer JA, Forstner R, Cunha TM, Kinkel K (2010). ESUR guidelines for MR imaging of the sonographically indeterminate adnexal mass: an algorithmic approach. *European Radiology*.

[7] Tamai K, Koyama T, Saga T (2006). MR features of physiologic and benign conditions of the ovary. *European Radiology*.

[8] Bazot M, Daraï E, Nassar-Slaba J, Lafont C, Thomassin-Naggara I (2008). Value of magnetic resonance imaging for the diagnosis of ovarian tumors: a review. *Journal of Computer Assisted Tomography*.

[9] Troiano RN, McCarthy S (1994). Magnetic resonance imaging evaluation of adnexal masses. *Seminars in Ultrasound CT and MRI*.

[10] Woodward PJ, Gilfeather M (1998). Magnetic resonance imaging of the female pelvis. *Seminars in Ultrasound CT and MRI*.

[11] Outwater EK, Mitchell DG (1994). Magnetic resonance imaging techniques in the pelvis. *Magnetic Resonance Imaging Clinics of North America*.

[12] Stevens SK, Hricak H, Campos Z (1993). Teratomas versus cystic hemorrhagic adnexal lesions: differentiation with proton-selective fat-saturation MR imaging. *Radiology*.

[13] Togashi K, Nishimura K, Kimura I (1991). Endometrial cysts: diagnosis with MR imaging. *Radiology*.

[14] Outwater EK, Dunton CJ (1995). Imaging of the ovary and adnexa: clinical issues and applications of MR imaging. *Radiology*.

[15] Low RN, Sigeti JS (1994). MR imaging of peritoneal disease: comparison of contrast-enhanced fast multiplanar spoiled gradient-recalled and spin-echo imaging. *American Journal of Roentgenology*.

[16] Stevens SK, Hricak H, Stern JL (1991). Ovarian lesions: detection and characterization with gadolinium-enhanced MR imaging at 1.5 T. *Radiology*.

[17] Siegelman ES, Outwater EK (1999). Tissue characterization in the female pelvis by means of MR imaging. *Radiology*.

[18] Balen A, Michelmore K (2002). What is polycystic ovary syndrome? Are national views important?. *Human Reproduction*.

[19] Heilbrun ME, Olpin J, Shaaban A (2009). Imaging of benign adnexal masses: characteristic presentations on ultrasound, computed tomography, and magnetic resonance imaging. *Clinical Obstetrics and Gynecology*.

[20] Kimura I, Togashi K, Kawakami S (1996). Polycystic ovaries: implications of diagnosis with MR imaging. *Radiology*.

[21] Tanaka YO, Tsunoda H, Kitagawa Y, Ueno T, Yoshikawa H, Saida Y (2004). Functioning ovarian tumors: direct and indirect findings at MR imaging. *Radiographics*.

[22] Kinkel K, Frei KA, Balleyguier C, Chapron C (2006). Diagnosis of endometriosis with imaging: a review. *European Radiology*.

[23] Bazot M, Thomassin I, Hourani R, Cortez A, Darai E (2004). Diagnostic accuracy of transvaginal sonography for deep pelvic endometriosis. *Ultrasound in Obstetrics and Gynecology*.

[24] Tanaka YO, Yoshizako T, Nishida M, Yamaguchi M, Sugimura K, Itai Y (2000). Ovarian carcinoma in patients with endometriosis: MR imaging findings. *American Journal of Roentgenology*.

[25] Gougoutas CA, Siegelman ES, Hunt J, Outwater EK (2000). Pelvic endometriosis: various manifestations and MR imaging findings. *American Journal of Roentgenology*.

[26] Outwater E, Schiebler ML, Owen RS, Schnall MD (1993). Characterization of hemorrhagic adnexal lesions with MR imaging: blinded reader study. *Radiology*.

[27] Kalish GM, Patel MD, Gunn MLD, Dubinsky TJ (2007). Computed tomographic and magnetic resonance features of gynecologic abnormalities in women presenting with acute or chronic abdominal pain. *Ultrasound Quarterly*.

[28] Bellah RD, Griscom NT (1989). Torsion of normal uterine adnexa before menarche: CT appearance. *American Journal of Roentgenology*.

[29] Rha SE, Byun JY, Jung SE (2002). CT and MR imaging features of adnexal torsion. *Radiographics*.

[30] Minutoli F, Blandino A, Gaeta M, Lentini M, Pandolfo I (2001). Twisted ovarian fibroma with high signal intensity on T1-weighted MR image: a new sign of torsion of ovarian tumors?. *European Radiology*.

[31] Thomassin-Naggara I, Dubernard G, Lafont C, Chopier J, Daraï E, Bazot M (2008). Imaging in pelvic inflammatory disease. *Journal de Radiologie*.

[32] Tukeva TA, Aronen HJ, Karjalainen PT, Molander P, Paavonen T, Paavonen J (1999). MR imaging in pelvic inflammatory disease: comparison with laparoscopy and US. *Radiology*.

[33] Kim SH, Yang DM, Kim KA (2004). Unusual causes of tubo-ovarian abscess: CT and MR imaging findings. *Radiographics*.

[34] Hawnaur JM, Reynolds K, McGettigan C (1999). Magnetic resonance imaging of actinomycosis presenting as pelvic malignancy. *The British Journal of Radiology*.

[35] Imaoka I, Wada A, Kaji Y (2006). Developing an MR imaging strategy for diagnosis of ovarian masses. *Radiographics*.

[36] Van Vierzen PBJ, Massuger LFAG, Ruys SHJ, Barentsz JO (1998). Borderline ovarian malignancy: ultrasound and fast dynamic MR findings. *European Journal of Radiology*.

